# Molecular Mechanisms of cGAS-STING Axis and Mitochondrial Dysfunction-Related Diseases in Humans: A Comprehensive Review

**DOI:** 10.2174/011570159X388747250830161230

**Published:** 2025-09-25

**Authors:** Xingtong Shen, Hantao Chen, Jishan Zheng, Yunyan Ma, Zhengzhen Tang, Hongqin Sun, Qian Zhang, Jidong Zhang, Tao Song

**Affiliations:** 1 Department of Immunology, Zunyi Medical University, Zunyi, China;; 2 Collaborative Innovation Center of Tissue Damage Repair and Regeneration Medicine, Zunyi Medical University, Zunyi, China;; 3 Special Key Laboratory of Gene Detection & Therapy of Guizhou Province, Zunyi Medical University, Zunyi, China;; 4 Department of Pediatrics, Third Affiliated Hospital of Zunyi Medical University, Zunyi, China

**Keywords:** cGAS-STING axis, mitochondrial dysfunction, molecular mechanisms, ischemia/reperfusion injury, autoimmune disorder, immune responses

## Abstract

Mitochondria play a critical role in immune cell differentiation, activation, and the regulation of innate immune responses. The cyclic GMP-AMP synthase (cGAS)-stimulator of interferon genes (STING) pathway is a key mediator of cytosolic DNA sensing and contributes to a broad spectrum of pathological processes, including infectious diseases, sterile inflammation, cancer, and autoimmune disorders. STING is activated in response to cytosolic DNA during infection and can restrict translation in RNA virus-infected cells as part of the innate immune response. Studies have shown that mitochondrial dysfunction, particularly the release of mitochondrial DNA (mtDNA), can act as a potent trigger of cGAS-STING signaling, linking mitochondrial damage to immune activation. Additionally, this pathway intersects with autophagy, metabolic regulation, and cell death mechanisms. This comprehensive review summarizes current advances in understanding the cGAS-STING axis and mtDNA release in the context of mitochondrial dysfunction, with a focus on their roles in disease pathogenesis and potential as therapeutic targets. We highlight recent progress in the development of targeted interventions and emphasize the importance of elucidating the regulatory mechanisms underlying STING activation in various pathological conditions, including neuroinflammation, cancer, ischemia/reperfusion injury, and autoimmune diseases.

## 1 INTRODUCTION

Despite significant progress in clinical treatments, many challenges remain in managing cancers, inflammatory diseases, ischemia/reperfusion injuries, and autoimmune disorders, among others [1, 2]. Mitochondria, the organelles responsible for energy production, play a critical role in regulating the innate immune system [3]. Additionally, mitochondrial function mediates the activation and differentiation of immune cells. Mitochondrial damage is also a significant contributing factor in many diseases [4, 5]. The STING pathway, an evolutionarily conserved signaling pathway found in vertebrates, is essential for antiviral immunity [6, 7]. Investigating the molecular mechanisms that underlie the release of mitochondrial DNA (mtDNA) due to mitochondrial dysfunction and its activation of the cGAS-STING pathway is crucial for developing potential treatments for these disorders.

The innate immune system recognizes and defends against foreign invaders *via* germline-encoded pattern recognition receptors (PRRs). During infection or inflammation, the pathogen-associated molecular patterns (PAMPs) and damage-associated molecular patterns (DAMPs) are sensed by PRRs that initiate an autonomous defense mechanism. There are several types of PRRs, such as Toll-like receptors (TLRs) [8], retinoic acid-inducible gene-1 (RIG-I)-like receptors (RLRs), nucleotide-binding oligomerization domain (NOD)-like receptors (NLRs), cytosolic DNA sensors, such as cGAS, absent in melanoma 2, and DNA-dependent activator of interferon (IFN) regulatory factors [9]. The dsRNA derived from dsRNA viruses was predominantly sensed by TLR3 from TLRs and RIG-I, melanoma differentiation-association gene 5 (MDA5) from RLRs, while nonself ssRNA was sensed by TLR7 and TLR8 [10, 11]. The recognition of PRRs activates the downstream signaling, thereby increasing the production of cytokines, chemokines, and IFN-I [12]. The STING protein plays a critical role in the immune response to cytosolic DNA [13], including both exogenous and endogenous double-stranded DNA (dsDNA), which is detected by the cGAS enzyme [14]. The cGAS-STING signaling pathway is an important DNA-sensing pathway involved in innate immunity and viral defense [6]. STING is primarily activated by cytosolic DNA during DNA virus infections; however, recent evidence suggests that mitochondrial dysfunction, or secondary signals from RNA virus infections, can also lead to the indirect activation of the cGAS-STING axis [15, 16]. It is important to note that many RNA viruses have evolved mechanisms to inhibit STING as a means of immune evasion [17]. This distinction is crucial, as STING is not simply “inhibited during RNA infection,” but rather, it can be modulated in more complex ways by viral proteins that suppress its activation as part of immune escape strategies [18, 19].

Mitochondria are energy-producing organelles that regulate the innate immune system [20]. In addition, mitochondria are involved in immune cell differentiation and activation [21]. Moderate mtDNA stress can enhance antiviral response and nuclear DNA repair *via* the cGAS-STING-IRF3 dependent pathway [22, 23]. However, excessive activation upon mitochondrial stress causes dysregulation of homeostasis, leading to an inflammatory response and autoimmune diseases. mtDNA was considered to be DAMPs released by injured cells to initiate an innate immune response *via* the activation of the cGAS-STING axis. A recent study demonstrated that Bax and Bak, two members of the Bcl-2 family with proapoptotic functions, were activated in cells undergoing apoptosis and regulated the growth of the apoptotic pore [24]. The mitochondrial inner membrane (MIM) herniates into the cytosol *via* the Bax/Bak macropores in the mitochondrial outer membrane (MOM), releasing mitochondrial matrix components, including mitochondrial DNA [25, 26]. The mtDNA leakage during injury or stress initiates the cGAS-STING signaling pathway and rapid production of IFNs [27]. Activated apoptosis suppresses this process [28]. However, Marco Tigano *et al.* observed that dsmtDNA released from mitochondria triggers the RIG-I-mitochondrial antiviral signaling protein (MAVS) signaling pathway as immune surveillance [29]. The voltage-dependent anion channel (VDAC) oligomer pores in MOM also allow mtDNA leakage and trigger cGAS-STING signaling, which is the underlying molecular mechanism of systemic lupus erythematosus (SLE) [30]. A recent study demonstrated that the mitochondria-associated vaccinia virus-related kinase 2 (VRK2) is involved in the formation of VDAC1 pores under stress [31]. Moreover, mitochondrial reactive oxygen species (mROS) produced *via* the electron transport chain also participate in the immune response through their interaction with hypoxia-inducible factor-1α (HIF-1α) and nuclear factor-κB (NF-κB) [32]. Thus, it is possible to combine mitochondrial damage with STING.

Upon activation by stimuli such as cytosolic DNA, cGAS undergoes a conformational change and produces 2’,3’-cyclic GMP-AMP (cGAMP) from ATP and GTP [33, 34]. The cGAMP induces the closure and rotation of the cytosolic C-terminal domain in STING, which contains four putative N-terminal transmembrane domains and a cytosolic C-terminal domain in the endoplasmic reticulum (ER) [5, 35]. Following the conformational change and subsequent translocation to the Golgi [36], tank-binding kinase 1 (TBK1) is recruited, which autophosphorylates and also phosphorylates STING at Ser365 *via* co-oligomerization [37, 38]. Then, phosphorylated TBK1 activates interferon regulatory factor 3 (IRF3) at the conserved pLxIS motif [39], which relies on the E3 ubiquitin ligase tripartite motif protein 32 (TRIM32) mediated-K63-linked ubiquitination of K224 [40, 41]. The E3 ubiquitin ligase ring finger protein 5 (RNF5) negatively regulates this process [42]. Next, the complex enters the

nucleus to promote the expression of interferon-stimulated genes (ISGs) and initiate the production of IFN-I [5, 43]. Furthermore, the phosphorylation of the inhibitory protein IκB by IκB kinase permits p50-RelA to reach the nucleus. Together with IRF50 in the nucleus, p3-RelA dimers induce the transcriptional production of IFN and other immunostimulatory genes [6]. STING activation causes NF‑κB activation and induces LC3 lipidation and promotes autophagosome formation *via* non‑canonical autophagic pathways [44]. STING is finally destroyed within the autophagosome and following the translocation from the Golgi to the lysosome [45, 46].

**Fig. (1):**
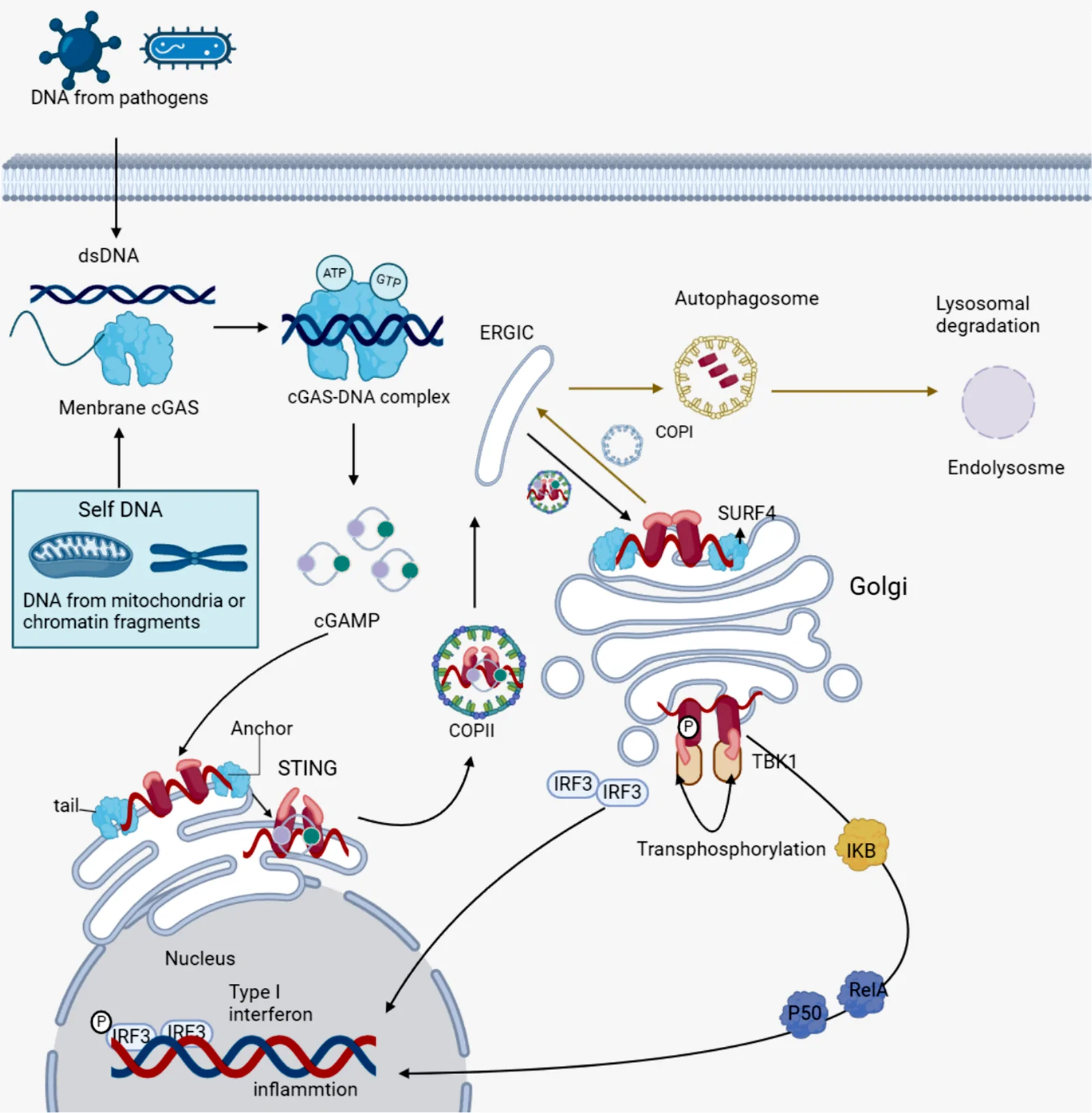
The cGAS-STING signaling pathway. Upon recognition of cytosolic dsDNA, either of pathogenic origin or released mtDNA resulting from mitochondrial dysfunction, cGAS is recruited to the DNA substrate, where it undergoes enzymatic activation and catalyzes the synthesis of the second messenger cGAMP.] cGAMP activates STING in the endoplasmic reticulum (ER). cGAMP activates STING in the ER, leading to changes in ER conception. STING then moves from the ER to the Golgi, where it activates TBK1/IKK/TBK1 to phosphorylate STING. STING subsequently recruits IRF3 for TBK1-mediated phosphorylation. Activated IRF3 dimerizes, penetrates the nucleus, and initiates IFN-I transcription, which causes an inflammatory response. IKK-mediated phosphorylation of IKB pro enables P50-Rela to translocate into the nucleus. STING activation can induce NF-κB activation and promote LC3 lipidation and autophagosome formation through non-canonical autophagy pathways. Finally, STING degradation takes place both within the autophagosome and from the Golgi to the lysosome in the absence of robust cGAMP stimulation. Additionally, STING translocation is inhibited by sequential retrograde translocation to the ER, which is mediated by COPI vesicles and facilitated by STING and SURF4 interactions.

This review investigates the cGAS-STING axis and mitochondrial dysfunction. It summarizes the pathogenesis and potential therapeutic strategies for cancers, inflammations, ischemia/reperfusion injuries (I/R), and autoimmune diseases (Fig. **1**).

## 2 METHODOLOGY

A comprehensive literature search was conducted using the PubMed database up to early 2025, without time restrictions. The search strategy involved Boolean combinations of terms including: ("cGAS-STING" OR "cyclic GMP-AMP synthase-stimulator of interferon genes") AND ("mitochondrial dysfunction" OR "mitochondrial diseases") AND ("neurodegeneration" OR "inflammation" OR "cancer" OR specific disease names).

Articles were included if they (1) focused on the role of the cGAS-STING pathway and mitochondrial function/dysfunction in human diseases, particularly studies with well-defined molecular mechanisms; and (2) comprised original research (basic or clinical) or high-quality reviews published in peer-reviewed journals.

Exclusion criteria included: (1) studies based solely on cellular or animal models without translational relevance to human diseases; (2) non-English publications; and (3) non-peer-reviewed content such as conference abstracts, editorials, or commentaries.

While a total of 173 articles were ultimately cited, a substantially larger number of studies were screened and evaluated for relevance and quality.

## 3 EFFECTS ON THE cGAS-STING AXIS AND MITOCHONDRIAL DYSFUNCTION IN DISEASES

### 3.1 Ischemia/Reperfusion Injury

Currently, understanding the mechanism of I/R injury is crucial to develop treatment strategies that can be applied in a clinical setting [47]. It commonly occurs in shock, sepsis, burns, and surgical procedures such as transplantation. Insufficient supply of oxygen and accumulation of toxins lead to cell necrosis. Moreover, the production of ROS and nitrogen species after reperfusion aggravates the inflammatory response, exacerbating organ injury and apoptosis [48]. Various studies have demonstrated that mitochondrial dysfunction plays a crucial role in I/R injury, and thus, this section discusses I/R injury and its association with STING. In intestinal I/R injury, disruption of the mucosal barrier integrity leads to cell death, bacterial translocation from the enteric cavity into circulation, and the massive release of DAMPs, which triggers the inflammatory response [49].

The mtDNA in the cytoplasm activates STING and elevates the production of IFN and tumor necrosis factor-α (TNF-α) by phosphorylating IRF3 and NF-κB. Then, IFN and TNF-α activate the receptor-interacting protein kinase 1 (RIPK1), which is a serine-threonine kinase component of the TNF receptor-1 signaling complex [49]. Upon deubiquitylated RIPK1 overexpression, RIPK3 is recruited, which interacts with RIPK1 *via* a homotypic RIP homology interaction motif [50]. Then, RIPK3 and RIPK1 form an amyloid-like signaling complex called the necrosome *via* autophosphorylation [51]. Downstream of this process, mixed-lineage kinase domain-like protein (MLKL) is phosphorylated by RIPK3, but not RIPK1, and undergoes oligomerization. Thereafter, MLKL is translocated to the plasma membrane, where it disrupts the permeability of the cell membrane by interacting with phosphatidylinositides [51]. Another study showed that renal I/R injury increases RIPK3 levels and promotes its translocation to the mitochondria, leading to aggravated renal injury. Subsequently, it interacts with mitofilin, an inner mitochondrial protein that can maintain the characteristic morphology of MIM [52], and RIPK3 triggers mtDNA release into the cytosol, which activates cGAS-STING-p65 axis to promote the production of proinflammatory cytokines/chemokines, such as IL-6, TNF-α, thereby worsening renal injury. Interestingly, the inhibitor of MLKL did not affect the mitofilin levels and cGAS-STING pathway, indicating that MLKL is not a mediator in this process [53]. In this way, we focus on another medium, the substances released by mitochondrial damage.

Typically, cGAS and STING collectively work in response to PAMPs or DAMPs, but they have individual effects on the disease process. Aging is a risk factor in I/R injury, and the mechanism of its involvement in aggravating liver I/R injury has been recently revealed. The nucleotide-binding domain and leucine-rich repeat-containing protein 3 (NLRP3) are activated in a STING-dependent manner that enhances the secretion of cytokines in aged mice and patients [54]. Interestingly, a study showed that cGAS, but not STING, is upregulated by oxidative stress in liver I/R injury. Elevated levels of cGAS can protect cytochrome c release from mitochondria and initiate autophagy to alleviate cell apoptosis [55].

Cerebral I/R injury is associated with the microglial cGAS-STING pathway. Microglia polarize into either the M1 or M2 form during I/R injury and neuroinflammation. M1 produces proinflammatory cytokines to aggravate injury, while M2 repairs damaged tissue with anti-inflammatory cytokines [56]. Thus, regulation of microglial polarization is pivotal for inflammation progression and treatment. STING and mtDNA are involved in microglial regulation. Upon I/R injury, mtDNA released into the cytosol triggers polarization of microglia to M1 by activating STING [57]. The occurrence of oxidative stress in I/R injury also enhances cGAS binding to pathogenic mtDNA. Histone deacetylases (HDACs) are also involved in cerebral I/R injury, wherein HDAC3 upregulates transcription of cGAS by deacetylating p65 and triggers its nuclear localization and transactivation, leading to the production of IFN [58]. Furthermore, the molecular processes behind STING axis dysfunction and mitochondrial damage offer avenues for future autoimmune disease therapies. These include inhibiting the STING signaling pathway [48], disrupting the interaction between RIPK3 and mitofilin [50], modulating NLRP3 activity [54], enhancing cGAS-mediated autophagy *via* small molecules [56], and employing HDAC3 inhibitors [58]. Collectively, these approaches offer novel therapeutic insights for the clinical management of I/R injury and associated autoimmune disorders.

### 3.2 Autoimmune Diseases

In the context of autoimmune diseases, immunity-related GTPases (IRGs) are critical, as they are part of the interferon-induced host resistance program and contribute to cell-autonomous immunity. Mouse IRGs are divided into two subfamilies: GKS, which serves as an “effector” during pathogen invasion by disrupting pathogen-containing vacuoles (PVs) and initiating antimicrobial processes [59-61] and GMS, which acts as a “regulator,” protecting self-structures by residing in endomembrane organelles such as mitochondria, the ER, and the Golgi apparatus [60]. In humans, IRGM binds to mitochondrial lipid cardiolipin, induces mitochondrial depolarization, and promotes mitochondrial fission in a manner dependent on leaky mtDNA, thereby regulating autophagy. Moreover, activation of IRGM elevates mROS [62], which can further contribute to immune dysregulation. The accumulation of DAMPs activates the cGAS-STING axis, playing a crucial role in autoimmune responses. It has been shown that mROS is not involved in mtDNA leakage [63], but IRGM acts as a major suppressor to prevent the overactivation of IFN-I in a p62-dependent manner. This suppression occurs through selective degradation of RIG-I and TLR-3 *via* the lysosomal pathway, and cGAS through both lysosomal and proteasomal pathways [64]. Recent studies also highlight that CSNK1A1/CK1α, a serine/threonine protein kinase, plays a critical role in preventing overactivation of IFN-I caused by STING1 through autophagic degradation of STING1, thus maintaining immune homeostasis [65]. Furthermore, the cGAS-STING pathway enhances lysosomal biosynthesis and activity independently of TBK1 by activating the transcription factor TFEB and inhibiting mTORC1-mediated phosphorylation of TFEB *via* STING-induced lipidation of GABARAP. This process aids in the clearance of cytoplasmic DNA and invasive pathogens, further influencing the pathogenesis of autoimmune diseases [66].

### 3.3 Systemic Lupus Erythematosus

Systemic lupus erythematosus (SLE) is a chronic multisystem autoimmune disease with multiple clinical manifestations, including the formation of auto-antibodies and immune complexes [67]. A study showed that SLE is characterized by prolonged IFN-α signaling that causes mtDNA accumulation in the cytoplasm, which is sensed by STING to induce the differentiation of monocytes into auto-inflammatory dendritic cells (DCs). IFN-α signaling results in mitochondrial stress, which leads to the deregulation of oxidative phosphorylation and increases the levels of mROS and ATP, thereby altering the lysosomal pH that affects efficient autophagy [68]. IRGM is a novel candidate gene that has been demonstrated to be associated with SLE. Gene-gene interaction between ATG5, ATG7, and IRGM confers susceptibility to SLE [69]. A recent study showed that the fever associated with patients with SLE is owing to the IRGM rs13361189 polymorphism [70]. Therefore, the link between the IRGM gene and mtDNA and STING in SLE patient needs to be further explored. Overall, therapeutic strategies for SLE can focus on targeting the IFN-α-mtDNA-STING axis, regulating autophagy, and modulating the function of the IRGM gene.

### 3.4 Multiple Sclerosis

Multiple sclerosis (MS) is an autoimmune disease of the central nervous system (CNS) characterized by chronic inflammation and neurodegeneration [71, 72]. While immunosuppressive drugs can modulate immune cell activity within the CNS, they are unable to prevent the progression of neurodegeneration [73, 74]. Inflammation and glutamate excitotoxicity are major contributors to neuronal death in MS [75]. STING functions as an inflammatory sensor in neurons, integrating interferon and glutamate-induced intracellular calcium signaling, inducing ferroptosis, and serving as a central regulator of MS-associated neurodegeneration [76, 77]. Notably, STING activation in neurons does not rely on the classical cGAS-STING pathway but rather on calcium-mediated transport of STING from the ER to the Golgi apparatus. Inhibition of STING alleviates disease symptoms and reduces neuronal loss in experimental autoimmune encephalomyelitis (EAE) mice, suggesting that STING could be a potential therapeutic target for MS-related neurodegenerative diseases [77, 78].

## 4 CANCER

Globally, cancer is widely researched in clinics and laboratories, and the underlying mechanism for evading immunosurveillance and immune escape from the innate immune system has been challenging scientists.

Immunotherapy is a strategy increasingly used for cancer therapy, indicating a new direction for antitumor immunity. Immunotherapy includes immune checkpoint inhibitors (ICIs), adoptive cell transfer (ACT), cancer vaccines, and so on [79]. Recent studies have shown that the STING-activating hydrogel (ZCCG) was developed by the coordination of Zn^2+^ and 4,5 imidazole dicarboxylic acid while integrating chitosan (a stimulator activated by the spike pathway) and CpG (an agonist of TLR9) to initiate and activate cGAS-STING and TLR9 pathway-mediated immunotherapy [80]. Furthermore, the nanotube-mediated mitochondrial transfer metabolic process involves mitochondria being transferred from immune cells to cancer cells, thereby enhancing their capabilities while depleting those of immune cells. Conversely, it has been found that the inhibition of nanotube construction dramatically decreases mitochondrial transfer and effectively avoids immune cell depletion. Nanotube-mediated mitochondrial hijacking is a potential novel target for developing the next generation of cancer immunotherapy agents [81]. SMPDL3A, a cGAMP-degrading enzyme generated by LXR-mediated lipid metabolism, has been demonstrated to limit cGAS-STING to DNA sensing, and pharmacological inhibition of SMPDL3A to boost cGAMP-STING immune responses may contribute to antiviral and anticancer immunity.

**Fig. (2):**
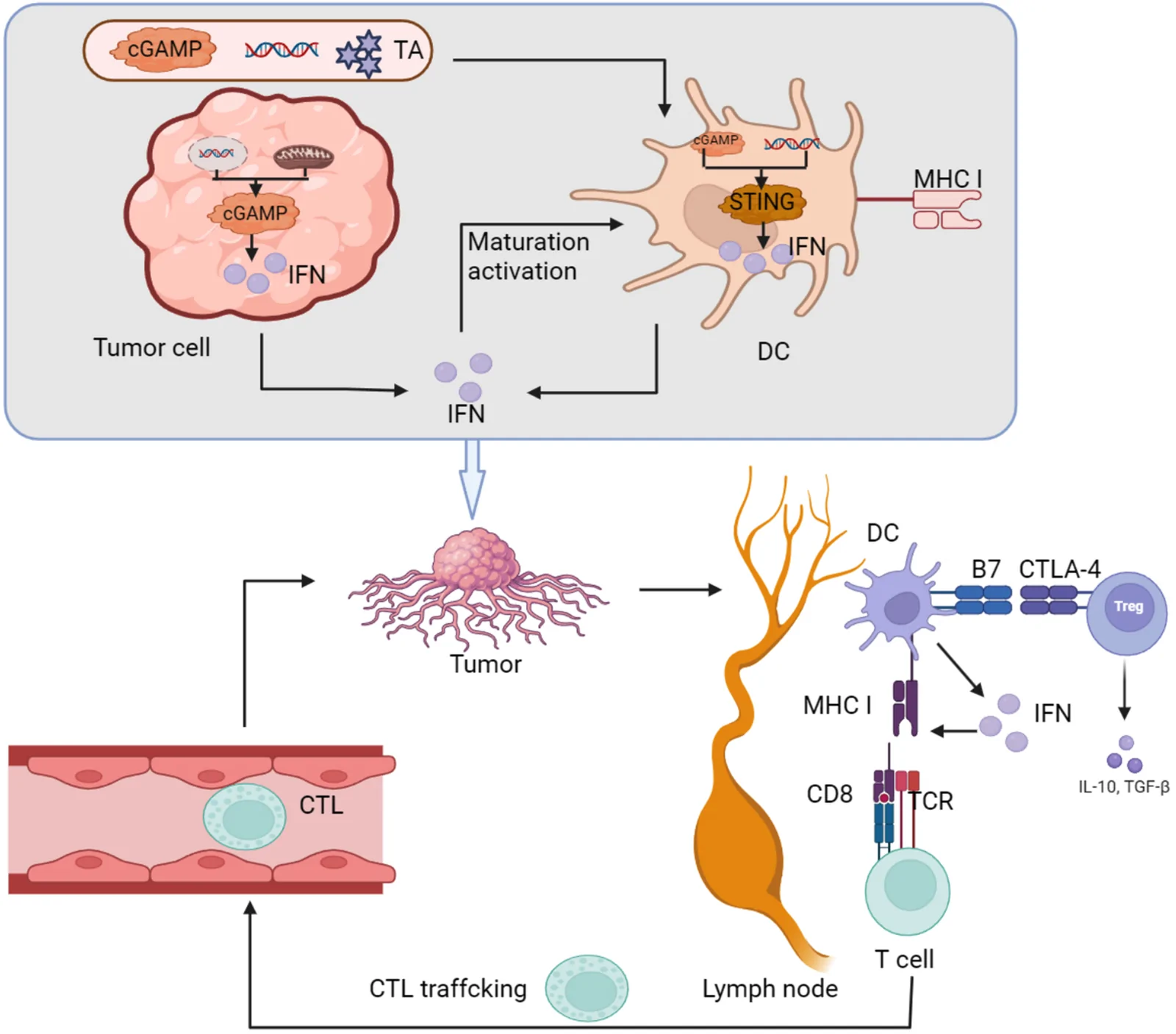
Schematic of the cGAS-STING axis in the cancer‑immunity cycle. Tumor‑derived DNA leakage into the cytosol is detected by cGAS, which synthesizes cGAMP to activate STING both in tumor cells and in neighboring DCs *via* direct DNA uptake or intercellular cGAMP transfer. In DCs, STING activation promotes antigen cross‑presentation and primes tumor‑specific CD8^+^ T cells. The resulting IFN‑I release mediates the majority of downstream immunostimulatory effects, driving effective antitumor immunity.

Immune escape is the underlying mechanism for the unlimited growth of cancer cells and metastasis. Some mechanisms are associated with STING and mitochondria. Mitochondrial Lon is a multifunctional protein that maintains mitochondrial homeostasis and plays a crucial role in the protein quality control system [82]. In the tumor microenvironment (TME), Lon-induced extracellular vehicles containing mtDNA and programmed cell death protein-1 (PD-L1) suppress T-cell function *via* initiating the secretion of immunosuppressive cytokines from M2 macrophages [83]. TME is a barren environment without ample nutrients for growth. The lack of available lipids within the TME enhances the expression of the fatty acid binding protein 5 (FABP5), a lipid chaperone for lipid metabolism, which is mainly expressed in Tregs and naive CD4^+^ T cells, but it cannot support the function of FABP5 and maintain mitochondrial metabolism [84]. In the TME, cGAS-STING in DCs is critical for tumor-specific CD8 T cell cross-presentation and initiation, and tumor-derived DNA can be picked up by DC-like protein antigens, leading to IFN-I upregulation, which is responsible for the majority of the biological effects of the cGAS-STING pathway on immune cells. However, beyond its anti-tumour immune effects, chronic or excessive STING activation also triggers immunosuppressive signals within the TME. Studies have demonstrated that sustained STING activation leads to the production of IL-10, the recruitment of regulatory T cells (Tregs), and the upregulation of IDO expression, all of which impair effector T cell function and promote tumour immune escape [85-87]. The defective mitochondrial metabolism leads to the disruption of mitochondria and mtDNA release, which triggers the cGAS-STING axis, prompting IFN expression, especially IL-10, and activating Tregs to achieve immunosuppression in TME [84] (Fig. **2**).

### 4.1 Lung Cancer

Lung cancer is one of the most common cancers found worldwide, with non-small cell lung cancer (NSCLC) accounting for a large proportion of lung cancers. The cGAS-STING axis and mitochondria are closely associated with antitumor immunity. The *KRAS* mutant is commonly observed in NSCLC, and the *KRAS* mutant with *LKB1* is aggressive as it lacks PD-L1 and poorly responds to immune checkpoint blockade. A study revealed that *LKB1* loss selectively suppressed STING expression in *KRAS-LKB1* mutant cells by enhancing the activity of DNMT1 and EZH2 to escape immunity recognition *via* sensing mtDNA leakage, leading to impaired intratumoral T cell recruitment and low expression of PD-L1 [88]. It highlighted the potential of STING agonists as an efficient treatment strategy. Few drugs have been identified for treatment or enhancing its efficiency to inhibit cancer cell growth. Rocaglamide, a natural product from *Aglaia odorata*, has been demonstrated to promote NK cell infiltration and enhance its antitumor immunity through mtDNA release and cGAS-STING activation, leading to increased expression of CLC5 and CXCL10 in NSCLC cells [89]. The derivatives of the natural alkaloid camptothecin (CPT), such as irinotecan and topotecan, have been used for chemotherapy in a clinical setting. However, lung cancer cells acquire chemoresistance, limiting its application for treatment. It was demonstrated that 4-[2,3,5,6-tetrafluoro-4-(4-hydroxyphenoxy) phenoxy] phenol (TFPP), a quinone-containing compound, promotes the capacity to inhibit the growth of NSCLC cells when used in combination with CPT by upregulating endogenous mROS release [90]. TFPP can also trigger CPT-induced apoptosis through mitochondria-mediated pathways by decreasing phosphor-ERK expression [90]. In summary, the combination of STING agonists, Rocaglamide, or TFPP with CPT therapy may offer a promising therapeutic approach for non-small cell lung cancer by modulating mtDNA release and activating the cGAS-STING axis.

### 4.2 Breast Cancer

Breast cancer is a leading cause of morbidity and mortality in women. Microtubule destabilizer eribulin causes the destabilization of the interphase microtubule network and accumulation of cytoplasmic mtDNA, which triggers expression of IFN-β and ISGs *via* cGAS-STING pathway in triple-negative breast cancer (TNBC) treatment. In contrast, eribulin can be used to treat TNBC by mediating the accumulation of cytoplasmic mtDNA, thereby activating the cGAS-STING pathway [91]. The cGAMP analog, cyclic di-AMP (c-di-AMP), promotes the translocation of IRF3 to the mitochondria and triggers apoptosis of cancer cells through the stabilization of STING [92]. Thus, the molecular mechanisms of STING and mitochondrial damage provide new ideas for clinical drug development in breast cancer. Therefore, the use of Eribulin and the cGAMP analogue c-di-AMP offers new insights into breast cancer treatment by modulating mtDNA release and activating the cGAS-STING axis.

### 4.3 Acute Myeloid Leukemia

**Fig. (3):**
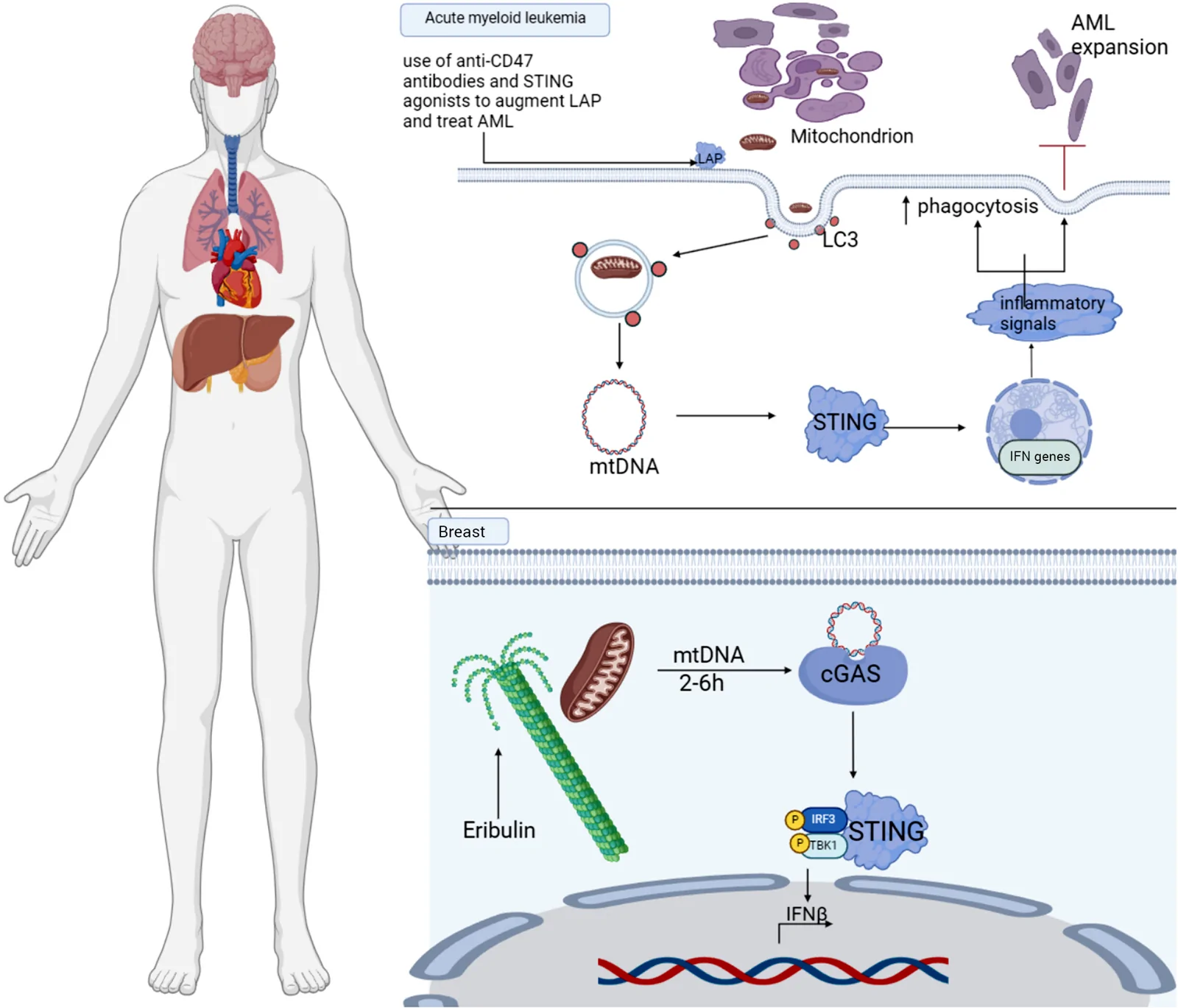
cGAS-STING activation in AML and breast cancer models. AML: Bone marrow macrophages clear mitochondria‑rich apoptotic vesicles from AML cells *via* LC3‑associated phagocytosis (LAP). The resulting mtDNA engages the cGAS-STING pathway, enhancing macrophage phagocytic activity and inhibiting residual AML cell proliferation. Breast cancer: Microtubule‑targeting agents (MTAs) induce release of mtDNA into the cytosol of tumor cells, activating the cGAS-STING pathway. When combined with immune checkpoint inhibitors, this innate immune activation may improve antitumor efficacy.

Acute myeloid leukemia (AML) is a heterogeneous disease associated with a wide range of molecular changes; therefore, long-term disease control requires multiple treatment methods. Among them, immunotherapy with T cell ICIs, including antibodies targeting newer targets, such as PD-1, PD-L1, CTLA-4, TIM3, and STING, can be used for treatment [93]. The mechanism involves the activation of the STING pathway *via* the LC3-associated phagocytosis (LAP) of acute AML-derived apoptotic bodies (ABs) by bone marrow-derived macrophages. Phenotypically, STING activation inhibited the growth of AML *via* increased phagocytosis [94] (Fig. **3**). Therefore, the use of immunotherapy, particularly T-cell immune checkpoint inhibitors (ICIs) targeting PD-1, PD-L1, CTLA-4, TIM3, and STING, presents new strategies for the treatment of AML.

## 5 INFLAMMATORY DISEASES

**Fig. (4):**
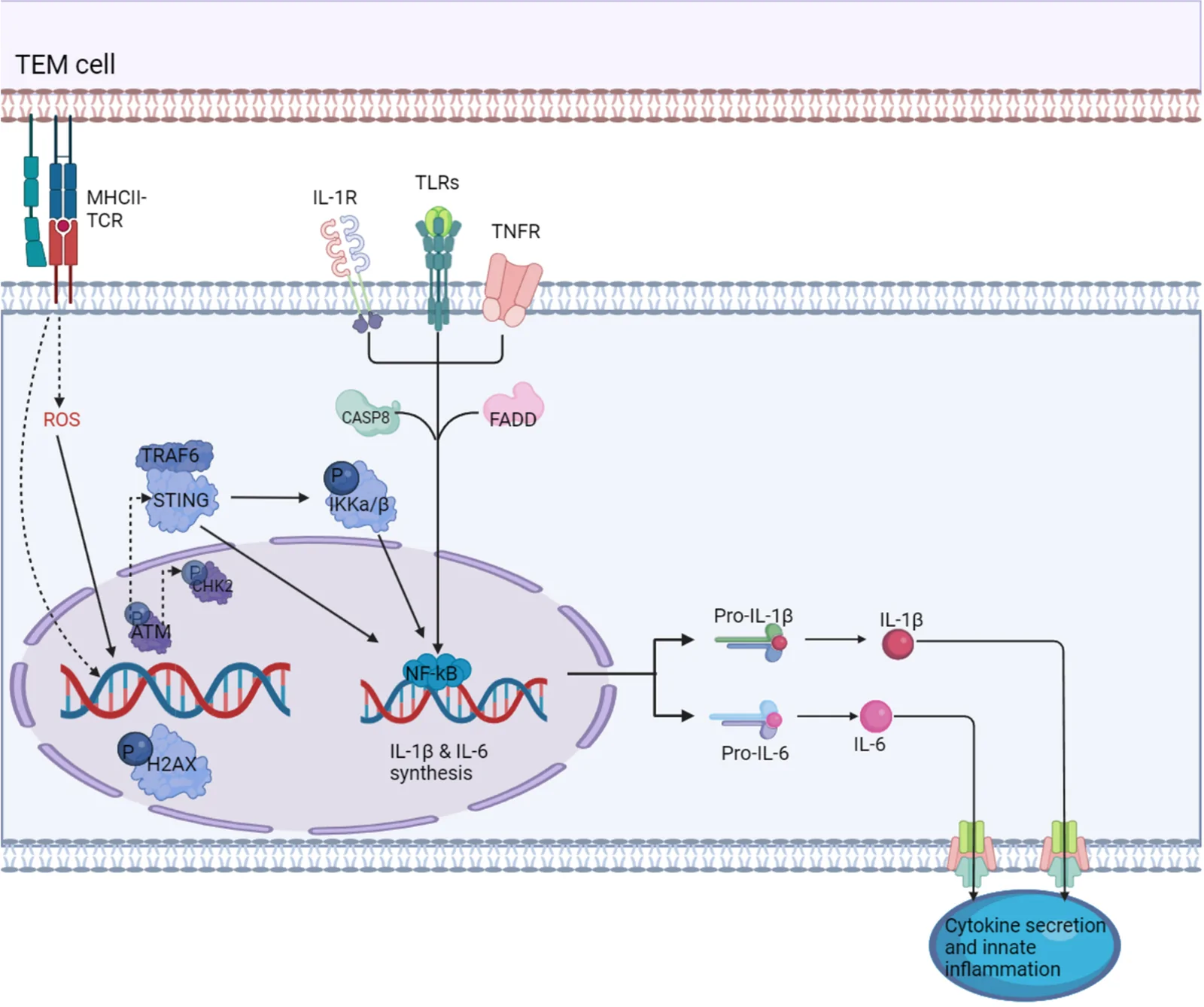
Models of cGAS-STING-mediated responses in host defense and neuroinflammatory diseases. Abbreviations: STING: interferon gene NF-κB: nuclear factor-activated κ-light chain enhancement in B cells; IL-1β: recombinant human interleukin-1β; IL-6: interleukin 6; TRAF6: TNF receptor-associated factor 6.

Inflammatory diseases are characterized by aberrant or chronic activation of the immune system in response to various internal or external stimuli, such as pathogens, toxins, or metabolic stress. A central pathological mechanism underlying these conditions is inflammation, which is commonly initiated by mitochondrial dysfunction. Studies have demonstrated that mtDNA release due to mitochondrial stress can activate the cGAS-STING pathway, contributing to inflammatory responses. Oxidized mtDNA can also bind to NLRP3 in the cytoplasm, promoting inflammasome activation [95]. These mechanisms are implicated in a range of inflammatory diseases, including neuroinflammatory disorders, cardiovascular inflammation, metabolic syndromes such as diabetes, as well as liver, kidney, and pulmonary diseases (Fig. **4**).

### 5.1 Neuroinflammatory Diseases

The cGAS-STING system reacts to foreign DNA (viruses and bacteria) and its own DNA (damaged DNA and mtDNA). Several studies have found that the cGAS-STING pathway is protective against bacterial and viral infections and also detrimental in several neuroinflammatory diseases. Effector memory T (TEM) cells interact with DCs in the presence of homologous major histocompatibility peptides and induce an innate inflammatory response. A recent study has demonstrated that the nonclassical STING pathway, involving interaction with TRAF6 and activation of NF-κB, can boost the transcription of IL-1 and IL-6. The oxidative stress-associated NF-κB pathway was enriched, mROS production radicalized the TEM cell-mediated DNA damage response process in DCs, and the absence of STING alleviated T cell-induced innate inflammatory responses [96]. The harmful role of this cGAS-STING-dependent IFN-I response in each of these disorders is discussed below.

### 5.2 Parkinson’s Disease

Parkinson’s disease (PD) is a neurological disorder characterized by classical motor features of Parkinsonism and complex nonmotor features. Parkinson's disease involves degeneration of dopamine neurons in the substantia nigra and deposition of eosinophilic cytoplasmic inclusion bodies containing α-synuclein known as Lewy bodies [97], and inflammation is a key factor associated with a number of neuroinflammatory diseases, including Parkinson's disease [98]. In addition, mitochondrial dysfunction also plays an important role in Parkinson's disease [99]. PTEN-induced putative kinase 1 (PINK1), a ubiquitin kinase, and parkin (PRKN), an E3 ubiquitin ligase, regulate mitophagy and contribute to PD etiology. Mutation in PINK1/PRKN leads to STING-mediated inflammation, and the loss of STING can alleviate this phenomenon [100, 101]. PRKN^−/−^ and PINK1^−/−^ mice demonstrated high levels of proinflammatory cytokines (IL-6 and IFN-β) and mitochondrial damage by exercise or mitochondrial mutation, but not at rest [100]. Interestingly, in a *Drosophila* study of PD, the loss of STING failed to suppress mitochondrial dysfunction and ameliorate Parkinsonism [102]. In addition to PINK1/PRKN, serum/glucocorticoid-related kinase 1 influences the glial microenvironment (astrocytes and microglia), leading to proinflammatory conditions that can also cause PD [103]. The depletion of VPS13C, a gene encoding a lipid transfer protein located at the contact sites between ER and late endosomes/lysosomes, leads to abnormal activation of the cGAS-STING pathway *via* lysosome dysfunction [104].

### 5.3 Amyotrophic Lateral Sclerosis

Amyotrophic lateral sclerosis (ALS) is a fatal and rapidly progressive neurodegenerative disease that is characterized by severe muscular atrophy and paralysis [105]. Patients with ALS demonstrate upper and lower motor neuronal loss in the brainstem and spinal cord. The transactive response DNA-binding protein 43 (TDP-43), a nuclear DNA/RNA-binding protein, accumulates in patients with ALS and translocates into mitochondria, where it induces the release of mtDNA through the mPTP. This subsequently activates the cGAS-STING pathway, resulting in increased expression of NF-κB and IFNs [106]. TDP-43 is also overexpressed in cardiovascular diseases. In addition to TDP-43, misfolded superoxide dismutase 1 also contributes to ALS pathogenesis by triggering mtDNA release and prompt production of IFN-I in microglia [107]. Neuronal connexin 36 and pannexin 1 were found to play a pivotal role in extending the inflammation to bystander cells. We therefore hypothesized that this could play a therapeutic role in combination with STING. These studies shed light on potential therapeutic strategies for ALS.

### 5.4 Other Neuroinflammatory Diseases

Inflammation in the nervous system is an important mediator in many diseases. Activation of the cGAS-STING pathway and subsequent IFN elevation was observed in traumatic brain injury (TBI), indicating that mtDNA-induced inflammation was a trigger for secondary injury/cell death in TBI [108]. In addition, STING also acts as a hydrogen ion channel mediating Golgi hydrogen ion efflux, which in turn mediates the activation of autophagic pathways and NLRP3 inflammatory vesicles. Of practical significance, the cell-permeable small molecule compound C53 can be used to isolate the interferon response of STING from its role in autophagy or inflammation [109].

Furthermore, both acute and chronic inflammation play a significant role in the development of fibrosis in benign airway stenosis (BAS). Recent studies suggest that following tracheal injury, dsDNA can activate the cGAS-STING pathway, which is crucial to the pathophysiology of BAS [110-112]. The fibrosis process is likely triggered by macrophages secreting IL-6, which subsequently activates STAT3 in fibroblasts [113]. Thus, the molecular mechanisms of sting and mitochondrial damage in other neuroinflammatory disorders that we have not mentioned above could also provide potential therapeutic targets.

### 5.5 Cardiovascular Inflammation and Diabetes

Economic growth and improvement in living standards have caused overnutrition that underlies several diseases worldwide. Currently, inflammation is increasingly believed to be a key promotor of lifestyle diseases.

#### 5.5.1 Atherosclerosis

For example, high-fat diet-induced cellular stress leads to mtDNA leakage, STING activation, IRF3 phosphorylation, and consequently, an inflammatory response in endothelial cells [114], which occurs in macrophages of vascular can be the cause of atherogenesis [115]. Overexpression of TDP-43 in macrophages enhanced mtDNA release and initiated the cGAS-STING-NLRP3 pathway, leading to an inflammatory response in diabetes patients.

Moreover, upregulation of lipid uptake in macrophages promotes scavenger receptor gene CD36 transcription *via* β-catenin and PPAR-γ complex regulation [116]. High levels of mROS under stress were associated with mtDNA release [117, 118]. Knockdown of autophagy-associated kinases, Akt2 and AMPK, can worsen the cardiac condition, indicating that autophagy participates in atherogenesis [119].

#### 5.5.2 Aortic Aneurysm and Dissection

Aortic aneurysm and dissection are two other common cardiovascular diseases with high morbidity and mortality. Under stress, cytosolic DNA present in smooth muscle cells elicits an inflammatory response *via* the cGAS-STING pathway along with matrix metalloproteinase-9 expression in macrophages [120]. Aging is another risk factor in aortic aneurysm, changing the proteome in aortic tissue [121], which can be protected by STING SNP R293Q [122].

#### 5.5.3 Diabetic Retinopathy

The occurrence of metabolic stress in diabetes is accompanied by progressive complications, such as diabetic retinopathy, prolonged wound healing, diabetic foot, and so on. Increasing evidence shows that mitochondria are involved in these processes, and metabolic stress affects the wound healing process in several ways. MtDNA release under stress activated the cGAS-STING-IRF3 axis and upregulated subsequent Hippo-Yes-associated protein (YAP), the effector of the Hippo pathway, which is a cellular repair signaling, inactivation *via* phosphorylated IRF3 binding to mammalian Ste20-like kinases 1 (MST1) promoter [123]. Inactivated YAP is associated with suppression of angiogenesis, which plays a pivotal role in wound healing. The occurrence of a high-glucose environment in diabetes results in impaired antioxidant capacity of mitochondria *via* accumulation of mROS in keratinocytes and subsequent initiation of inflammation and promotion of apoptosis [124]. The ERK1/2-PI3-K/Akt-tuberin-mTOR pathways activated the cGAS-STING-IRF3 axis in keratinocytes for wound healing, which also had an effect on the retina [125]. STING-IRF3 is involved in islet β-cell lipotoxicity in type 2 diabetes mellitus(T2DM) [126]. Additionally, STING-IRF3 can also be induced in the skin of patients with T2DM to aggravate psoriasis [127].

#### 5.5.4 Obesity

Lifestyle-related diet can also induce thermogenesis. Brown and beige adipose tissue are two key participants in metabolism and thermogenesis owing to their high density of mitochondria. Obesity-related diet inhibited disulfide-bond A oxidoreductase-like protein, a chaperone-like protein found in mitochondria associated with mitochondrial integrity [128], triggering mtDNA leakage in brown adipose tissue and activating the cGAS-STING pathway. Initiation of this pathway decreases cAMP levels and PKA signaling *via* phosphodiesterase PDE3B/PDE4 activation, leading to impaired thermogenesis [129]. It was revealed that depletion of thioredoxin-2, a scavenger of mROS, enhances the inflammation in adipose tissue *via* the cGAS-STING-NLRP3 inflammasome pathway (especially NLRP3), indicating that it is another mollicute that evolves in metabolic regulation [130].

Recent research has revealed the presence of the mitochondrial melatonergic pathway in various body and brain cells [131, 132], with the NF-kB induction of an M1 phenotype in macrophages [133] and microglia [134] sequentially upregulating melatonin production that has autocrine effects to switch M1 phenotypes to M2 phenotypes. Different components of the NF-κB dimer can have differential effects, with p50/p50 suppressing the melatonergic pathway, whilst RelA and c-Rel can increase the melatonergic pathway [134]. Melatonin efflux also has paracrine effects, highlighting the complexity of alterations in the homeostatic interactions that all cells have in their given microenvironments, including in the pathoetiology of autoimmune disorders. As melatonin is a significant optimizer of mitochondrial function, partly *via* the upregulation of sirtuin-3 and associated increase in OXPHOS and decrease in mitochondrial electron transport chain oxidant production [135, 136], the regulation of the melatonergic pathway, including by different NF-κB dimers, will be an important avenue for future investigation on how cGAS-STING is integrated with wider core cellular processes relevant across a host of diverse medical conditions, especially given the role of melatonin in cGAS-STING regulation [137, 138]. This would also clarify the role of the mitochondrial melatonergic pathway in the modulation of mitochondrial ROS induction of mtDNA release. The role of the melatonergic pathway in the tumor microenvironment has been recently reviewed [139]. It will also be important for future research to clarify the role of increased methylglyoxal in diabetes, obesity and metabolic syndrome in the pathoetiology of a host of medical conditions, given data showing methylglyoxal to regulate cGAS-STING [140].

The four categories of diabetes-related cardiovascular disease-atherosclerosis, Aortic aneurysm and dissection, Diabetic retinopathy, and obesity-are discussed in these four paragraphs. Furthermore, as inflammation is a common feature in all of these illnesses, studies understanding the molecular origins of tingling and mitochondrial damage ought to produce novel strategies for treating patients.

### 5.6 Liver Disease

The X-box binding protein 1 (XBP1), a primary component of ER stress, is suppressed in acute liver injury, leading to impairment of mitophagy, which could be reversed by overexpression of PINK1 in hepatocytes. This leads to the upregulation of mROS and leakage of mtDNA. The production of mROS triggers hepatocellular pyroptosis *via* the NLRP3/caspase-1/GSDMD pathway. Meanwhile, leaked mtDNA engulfed by macrophages initiates the STING pathway [141]. A study on aged macrophages revealed a decline in PINK1/PRKN-mediated mitochondrial polyubiquitination and lysosome formation and function *via* the mTOR/transcription factor EB pathway, leading to mtDNA release and STING activation. Conversely, overexpression of PINK1 could only ameliorate impaired mitochondrial ubiquitination but not lysosome formation, which could be reversed by Torin-1 treatment [142]. With the exception of pyroptosis, activation of IRF-3 as well as STING was observed in ethanol-induced alcohol liver disease under ER stress, which triggered hepatocyte apoptosis independent of inflammation [143]. Therefore, exploring the above molecular mechanisms also provides new therapeutic targets for the treatment of liver disease.

### 5.7 Kidney Diseases

Mitochondrial damage and mtDNA leakage through BAX were observed in cisplatin-induced acute kidney injury (AKI), leading to inflammation *via* the cGAS-STING signaling pathway [144]. A novel STING antagonist, H151, ameliorated the symptoms of AKI [145]. Notably, mitochondrial dysfunction upon AKI impaired mitochondrial homeostasis, including downregulation of mitochondrial transcription factor A. This downregulation was associated with decreased expression of ATP synthesis genes, decreased mitochondrial DNA copy number, impaired electron transport chain, and mature collagen deposition, which is a hallmark of fibrosis [146, 147]. Depletion or knockdown of STING improved the inflammatory response in kidney diseases.

### 5.8 Pulmonary Diseases

In a lipopolysaccharide-induced acute lung injury (ALI) mouse model, cytosolic mtDNA and transcription factor c-Myc together upregulated STING expression, leading to enhanced pyroptosis of macrophages *via* NLRP3 [148]. Thus, augmentation of STING expression triggered an inflammatory response in the lungs and extrapulmonary organs, leading to deterioration. STING-induced impaired autophagy by cytosolic mtDNA was shown to be involved in this process by affecting lysosomal acidification and, subsequently, the digestion rate in macrophages, which is a turning point for deterioration [149]. COVID-19, characterized by severe and progressive ALIs, is associated with cytokine storms owing to mtDNA leakage-induced STING activation in macrophages [150]. Typically, mtDNA leakage during stress or disruption is detected by cGAS, which subsequently activates STING.

Metabolic reprogramming in macrophages plays a pivotal role in the inflammatory response. HIF-1α stabilization was increased *via* a STING-induced mROS release in the M1-like macrophages, leading to increased glycolysis and decreased oxidative phosphorylation, with subsequent production of IFN-β [151]. Conversely, both glycolysis and mitochondrial respiration were decreased in murine bone marrow-derived macrophages infected by live *Mycobacterium tuberculosis* (*Mtb*) in an IFN-I-dependent manner, which can be reversed by the knockdown of the IFN receptor or the upstream regulator STING [152]. The virulence and viability of *Mtb* is strain-dependent [153]. With the exception of *Mtb*, rough type-*Mycobacterium abscessus* (Mab-R) is also a tricky problem that leads to pulmonary infection. Induction of mROS plays a pivotal role in MAB-R infection, which can enhance IFN-I production in a mtDNA-activated STING manner and IL-1β production *via* NLRP3 inflammasome activation in macrophages. IL-1β is capable of antibacterial effects, but IFN-I production activates NLRP3 inflammasome *via* the STAT-I signal, which contributes to enhanced bacterial survival [154]. Moreover, activation of bacterial replication occurs in MAB-R injected macrophages *via* phagosomal rupture and bacterial escape to the cytosol in an IFN-I-dependent manner [155], while that of *Mtb* occurs *via* ESX-1-mediated phagosomal rupture [156]. Additionally, ER stress also occurs in *Mycobacterium* infection, where the levels of transcription factor XBP-1 were increased during infection with *Mycobacterium bovis,* which is associated with the activation of the STING-TBK1-IRF3 pathway [157].

Another microorganism that commonly causes lung infection is *Streptococcus pneumoniae* (*S. pn*). *S. pn* secretes hydrogen peroxide (H_2_O_2_), which causes mitochondrial oxidative stress, leading to mtDNA damage and leakage, followed by IFN-β production in the alveolar epithelial cells [158]. Another pathogenic factor, pneumolysin, also contributes to oxidative stress in macrophages [159]. A study demonstrated that *Streptococcus pneumoniae S. pn* M5 protein enhances the production of IL-10, which is the downstream effect of STING-induced IFN-I activation in a cGAS-independent manner [160]. However, more evidence is required to confirm the interaction between IL-10 and IFN-I activation.

STING was activated in silica-induced lung disease in mice, leading to IFN-I response *via* mtDNA as well as nuclear DNA release in a cGAS-independent manner, but involved the upregulation of DNA sensors, such as DDX41 and IFI204. DNase I treatment alleviated this inflammatory response in mice [161]. Moreover, other disease-induced lung injury has been demonstrated to be associated with STING, including burn-induced lung injury [162] and scleroderma-associated interstitial lung diseases [163]. Furthermore, STING and other inflammatory mediators were activated in stable chronic obstructive pulmonary disease, irrespective of the severity, which is considered a protective mechanism for sensing potential infection [164] (Table **1**).

## 6 DISCUSSION AND PERSPECTIVE

The relationship between the cGAS-STING axis and mtDNA release induced by mitochondrial dysfunction has generated immense interest among researchers in recent years. As previously noted, cGAS-STING functions as a key DNA-sensing mechanism in innate immunity and virus defense [6], and mitochondria also contribute to immune cell differentiation and activation [21]. There is compelling evidence that the relationship between the cGAS-STING axis and mtDNA release caused by mitochondrial dysfunction may provide potential treatment strategies for several diseases, including cancer, inflammation, I/R injury, and autoimmune diseases, although further research is required to validate the current findings.

Mitochondria are crucial organelles involved in ATP production and stress response through autophagy. Recent studies have discovered that mitochondrial dysfunction plays a role in the development of several diseases, and it is not limited to genetically related disorders such as Leber hereditary optic neuropathy or chronic progressive external ophthalmoplegia. This dysfunction is attributed to the release of mitochondrial nucleic acids. Due to their bacterial ancestry, mitochondrial nucleic acids possess the ability to elicit a reaction, such as in circumstances when present outside the mitochondria, by interacting with certain sensors and signaling pathways within the innate immune system. However, it is imperative to conduct further validation, investigate additional potential mechanisms, and establish correlations with systemic pathology. This will enable us to acquire a comprehensive understanding of the prerequisites and underlying factors associated with cGAS-STING-driven therapy for various diseases, as well as the activation of mitochondrial extracellular responses through specific pathways. Despite the current advancements, there are several unresolved queries that necessitate attention. For instance, it has been observed that targeted reprogramming of tumor-associated macrophages holds promise as a therapeutic approach for solid tumors [165]. However, the precise interplay between IL-10 and IFN-I activation in the metabolic reprogramming of macrophages regulated by STING remains unclear. Moreover, the underlying mechanisms of these findings are still not completely understood, thereby necessitating further investigation. It is understood that the accumulation of mROS within keratinocytes results in the reduction of mitochondrial antioxidant capacity. Consequently, this triggers inflammation and facilitates cellular apoptosis. Phosphorylation of IRF3 and NF-κB occurs in the cytoplasmic mtDNA environment and utilizes the STING pathway to enhance the production of IFN and TNF-α. *In vitro* studies have shown that STING activation is associated with inducing T-cell stress and apoptosis [166]. Typically, during the stress or disruption process detected by cGAS, mtDNA leaks into the cytosol, followed by STING activation. Activation of STING is known to regulate mitochondrial membrane pores, autophagy, and mPTP. The STING pathway is increasingly known to have other antitumor functions in the host’s immune response to tumors [167]. It is apparent that cGAS-STING stimulation of the immune system may be a valuable treatment technique for early and chromosomal stable cancers; however, caution should be exercised as inhibition of this route based on STING agonists may lead to drug resistance and unexpected fragility. The current understanding of cGAS-DNA interactions remains limited, particularly regarding the variations in cGAS binding affinity toward distinct ligands. Another area of interest is the interaction between cGAS and various forms of chromatin, specifically, to examine whether nuclear cGAS causes the production of cGAMP, the circumstances under which this occurs, and the specific characteristics that render chromatin within the micronucleus a cGAS agonist [5]. The induction of the STING pathway within the TME has exhibited significant antitumor effects in experimental models. Nevertheless, the reason for the absence of similar outcomes in the clinical context remains uncertain. Furthermore, it is crucial to explore strategies to enhance the therapeutic effectiveness of appropriate STING activation in humans [168]. Furthermore, the targeted suppression of the cGAS-STING pathway in certain types of cancers can decrease the efficacy of STING agonist therapy. The cGAS-STING signaling activation or inhibition may also be beneficial in the treatment of several disorders, thereby warranting additional investigation.

Recent studies have expanded the functional landscape of the cGAS-STING pathway beyond innate immunity, highlighting its role in cellular senescence and age-related inflammation [169]. Mechanistically, cGAS-STING promotes formation of the IRF3-RB-E2F complex, maintaining RB in a hypophosphorylated state to induce senescence in hepatic stellate cells and limit fibrosis [170, 171]. In neurodegenerative models, aberrant cGAS-STING activation in senescent astrocytes drives pathological SASP *via* the LCN2-YY1 axis, while cGAS deficiency alleviates neurodegeneration [172]. Additionally, impaired YAP/TAZ signaling in aged stromal cells enhances cGAS-STING-mediated inflammation, and co-targeting these pathways may mitigate senescence-associated dysfunction [173]. These mechanisms are increasingly recognized as relevant to both neurodegenerative diseases and cancer. Overall, these findings broaden our understanding of cGAS-STING signaling and suggest that its modulation may offer novel therapeutic opportunities for fibrosis, neurodegeneration, and other aging-related disorders.

## STUDY LIMITATION

Although increasing evidence highlights the critical interplay between mitochondrial dysfunction, mtDNA release, and cGAS-STING signaling, several limitations should be acknowledged. Most available evidence comes from *in vitro* experiments or animal studies, which may not fully capture the complexity of human physiology and disease. Moreover, the heterogeneity of experimental systems and disease contexts hinders the establishment of a unified framework for interpreting the role of cGAS-STING activation across different pathological conditions. In addition, the long-term safety and efficacy of pharmacological modulation of this pathway remain uncertain, raising concerns regarding potential off-target effects and the risk of therapeutic resistance. Finally, robust clinical investigations are still lacking, and further studies are essential to validate the translational relevance of preclinical findings. Addressing these limitations will be crucial for the safe and effective development of cGAS-STING-targeted therapies.

## CONCLUSION

(1) The potential mechanisms linking cGAS-STING and mitochondrial dysfunction.

Mitochondrial dysfunction is a key factor in activating the cGAS-STING pathway through several mechanisms, including mtDNA leakage *via* membrane pores (such as Bax/Bak, VDAC, and mPTP), excessive production of mROS, and impaired autophagy. These processes result in the release of mtDNA into the cytosol, triggering cGAS-STING signaling and amplifying inflammatory responses. Furthermore, defects in mitophagy, particularly through the PINK1/Parkin pathway, contribute to mitochondrial dysfunction and STING-driven inflammation. ER-mitochondria crosstalk, inflammatory cell death pathways like necroptosis and pyroptosis, metabolic reprogramming in the tumor microenvironment, and pathogen-induced mitochondrial stress also enhance cGAS-STING activation. Additionally, genetic and epigenetic factors, such as STING polymorphisms and epigenetic silencing, influence immune responses and disease susceptibility. Targeting these pathways, including the use of STING modulators or mitophagy enhancers, holds significant potential as therapeutic strategies to mitigate diseases driven by mitochondrial dysfunction.

(2) Therapeutic Implications and Future Directions of Targeting the cGAS-STING-Mitochondria Axis.

In summary, the cGAS-STING pathway and mitochondrial dysfunction-induced mtDNA release represent critical mechanisms with therapeutic potential across cancer, inflammation, ischemia/reperfusion injury, and autoimmune diseases. While cGAS-STING activation has shown promise in enhancing immune responses—particularly in oncology—further studies are needed to clarify its regulatory mechanisms and optimize clinical applications.

Emerging evidence suggests that redox-dependent hormetic stress and the Nrf2-associated resilience network, including vitagenes such as HO-1 and heat shock proteins, may intersect with mitochondrial signaling and cGAS-STING-driven immunity. This highlights a potential link between redox homeostasis and immunometabolic reprogramming, offering new directions for therapeutic refinement. Further investigation into these interconnected pathways will be essential for improving the efficacy and safety of STING-targeted interventions.

## Figures and Tables

**Table 1:** Mechanistic interplay between STING pathway activation and mitochondrial dysfunction in human diseases.

**Types of Disease** **or Condition**	**Specific Diseases**	**Pathways Related to STING and Mitochondrial Dysfunction**	**References**
Ischemia/reperfusin injury	Ischemia/reperfusin injury	STING and mtDNA involved in microglial regulation.	[47-58]
Autoimmune diseases	Autophagy	Human IRGM binds to the mitochondrial lipid cardiolipin, induces depolarization, and promotes mitochondrial fission to regulate autophagy in a leaky mtDNA-dependent manner. CSNK1A1 in maintaining immune homeostasis by autophagic degradation of STING1.	[59-66]
	Systemic lupus erythematosus (SLE)	lupus characteristic IFN-α leads to cytoplasmic accumulation of mtDNA and sensed by STING to activate monocytes differentiation into autoinflammatory dendritic cells (DCs).	[67-70]
Cancer	Lung cancer	LKB1 loss selectively suppressed STING expression in KRAS-LKB1(KL) cell ,leads to impair intratumoral T cell recruitment.	[88-90]
	Breast	Accumulation of cytoplasmic mtDNA triggers expression of interferon-β and ISGs *via* the cGAS-STING pathway.	[91, 92]
	Acute myeloid leukemia	STING activation can inhibit the growth of AML through the mechanism related to the increase of Phagocytosis.	[93 ,94]
Inflammation	Neuroinflammatory diseases	Non-classical STING pathway, through interaction with TRAF6 and activation of the transcription factor NF-kB,can boost IL-1 and IL-6 transcription.	[96]
	Parkinson’s disease	Mutation in PINK1/Parkin leads to STING-mediated inflammation and the loss of STING can alleviate the phenomenon.	[97-104]
	Amyotrophic lateral sclerosis	TDP-43 leads to mtDNA release through the mitochondrial permeability transition pore (mPTP) which elevated NF-κB along with interferons *via* cGAS-STING pathway.	[105-107]
	Other neuroinflammatory diseases	STING acts as a hydrogen ion channel mediating Golgihydrogen ion efflux, which in turn mediates the activation of autophagic pathways and NLRP3 inflammatory vesicles.	[108-113]
	Metabolic stress in cardiovascular inflammation and diabetes	Overexpression of TDP-43 in macrophages enhancing mtDNA release and initiating cGAS-STING-NLRP3 pathway leading to inflammatory response.	[114-140]
	Liver disease	Production of mtROS trigger hepatocellular pyroptosis *via* NLRP3/caspase-1/GSDMD pathway meanwhile leakage mtDNA engulfed by macrophage initiate STING pathway.	[141-143]
	Kidney diseases	Mitochondria damage and leakage mtDNA through BAX was found in cisplatin-induced acute kidney injury (AKI) leading to inflammation *via* cGAS-STING signaling.	[144-147]
	Pulmonary diseases	Phosphorylation and the expression of STING were respectively upregulated by cytosolic mtDNA and transcription factor c-Myc as well as the enhancing pyroptosis through NOD-like receptor protein 3 (NLRP3).	[148-164]

## LIST OF ABBREVIATIONS

ABs: Apoptotic Bodies
ACT: Adoptive Cell Transfer
AIM2: Absent in Melanoma 2
AKI: Acute Kidney Injury
ALIs: Acute Lung Diseases
ALS: Amyotrophic Lateral Sclerosis
AML: Acute Myeloid Leukemia
ATG: Autophagy-related
BMDMs: Bone Marrow-derived Macrophages
c-di-AMP: Cyclic di-AMP
cGAMP: 2’,3’-cyclic GMP-AMP
cGAS: Cyclic GMP-AMP Synthase
COEP: Chronic Progressive External Ophthalmoplegia
CPT: Camptothecin
DAI: DNA-dependent Activator of IFN Regulatory Factors
DAMPs: Damage-associated Molecular Patterns
DCs: Dendritic Cells
DsbA-L: Disulfide-bond A Oxidoreductase-like Protein
ER: Endoplasmic Reticulum
ETC: Electron Transport Chain
EVs: Extracellular Vesicles
FABP5: Fatty Acid Binding Proteins 5
HDACs: Histone Deacetylases
HIF-1α: Hypoxia-inducible Factor-1α
I/R: Ischemia/Reperfusion
ICB: Immune Checkpoint Blockade
ICIs: Immune Checkpoint Inhibitors
IFN: Interferon
IRF3: Interferon Regulatory Factor 3
IRGs: Immunity Related GTPases
ISGs: Interferon Stimulated Genes
KL: KRAS-LKB1
LAP: Related Phagocytosis
MAB-R: *Mycobacterium abscessus*, Resistant Strain
MIM: Mitochondrial Inner Membrane
MLKL: Mixed-lineage Kinase Domain-like Protein
MN: Motor Neuronal
MOM: Mitochondrial Outer Membrane
mPTP: Mitochondrial Permeability Transition Pore
MST1: Mammalian Ste20-like Kinases 1
*Mtb*: *Mycobacterium Tuberculosis*
mtDNA: Mitochondrial DNA
mtROS: Mitochondrial Reactive Oxygen Species
NF-κB: Nuclear Factor-κB
NLRP3: Nucleotide-binding Domain and Leucine-rich Repeat Containing Protein 3
NOD: Nucleotide-binding Oligomerization Domain
NSCLC: Non-small Cell Lung Cancer
Ox-mtDNA: Oxidized Mitochondrial DNA
PAMPs: Pathogen-associated Molecular Patterns
PD: Parkinson’s Disease
PINK1: PTEN-induced Putative Kinase 1
Ply: Pneumolysin
PRRs: Pattern Recognition Receptors
PV: Pathogen-containing Vacuole
RHIM: RIP Homology Interaction Motif
RIG-I: Retinoic Acid-inducible Gene-1
RIPK1: Receptor-interacting Protein Kinase 1
RNF5: Ring Finger Protein 5
ROS: Reactive Oxygen Species
SGK1: Serum/Glucocorticoid Related Kinase 1
SLE: Systemic Lupus Erythematosus
STING: Stimulator of Interferon Genes
T2DM: Type 2 Diabetes Mellitus
TAMs: Tumor-associated Macrophages
TBK1: Tank-binding Kinase 1
TDP-43: Transactive Response DNA-binding Protein 43
TEM: Effector Memory T
TFAM: Transcription Factor A
TFPP: 4-[2356-tetrafluoro-4-(4-hydroxyphenoxy)phenoxy]phenol
TLRs: Toll-like Receptors
TM: Transmembrane
TME: Tumor Microenvironment
TNBC: Triple-negative Breast Cancer
TNF: Tumor Necrosis Factor
TRIM32: Tripartite Motif Protein 32
TRX2: Thioredoxin-2
VDAC: Voltage‐dependent Anion Channel
VRK2: Virus-related Kinase 2
YAP: Yes-associated Protein
ZCCG: 2+and 4,5imidazoledicarboxylic Acid of Hydrogel
